# The Serum of Sobriety

**Published:** 1899-05

**Authors:** 


### THE SERUM OF SOBRIETY.

THE genial editor of *The British Medical Journal*, in a recent sportive mood, makes fun of the progressive spirit of some of his transatlantic brethren. He does it, however, in a jocular spirit which even the American can appreciate and enjoy. Hear what he says:

"If any one wished to embody the most striking feature of the therapeutic 'movement' of the day in a classic formula, he would —if he cared to use the license of the 'higher criticism' and give the words a meaning undreamed of by the author—find one in Ovid's phrase, *Sero medicina paratur*. In our battles against disease we conquer in the sign of Serum. Such virtue has this elixir that by means of it—if we are to believe the prophets of the New Medicine—we can make ourselves 'immune' against the invisible foes which go up and down the world seeking whom they may devour—including, it may be presumed, the 'microbe of death,' not long ago run to ground in a transatlantic laboratory. But this is not all. Serum, it appears, if the right 'brand' be used, will also 'immunize' us against moral disease. For instance, if we wish to render ourselves proof against inebriety, so that even after taking the pledge we shall feel no temptation to subdue our inordinate sense of our own virtue by a corrective dram, we need only submit to a few injections of a serum prepared by Dr. Evelyn, of San Francisco. This serum is extracted from a horse previously made suitable for the purpose by a course of alcohol. The noble animal has from two to fifteen pints of whisky administered to him daily for three months. At the end of that time a serum can generally be obtained which, inoculated into the most confirmed toper, at once leads him to ask for a 'blue ribbon.' The serum is preventive as well as curative; we are told that a child immunized with it is protected against drunkenness for the rest of his days. Thus may the virtue of temperance inoculate our old stock and Dr. Dawson Burns find his occupation gone. Dr. Evelyn calls his precious serum 'equisine.' We venture to suggest that he might next turn his attention to the treatment of folly. He could doubtless extract an appropriate serum from an animal nearly related to the horse. There is a large field for the therapeutic use of 'asinine' at the present day, and the ingenious American physician might begin with the patients who have been through a course of his 'equisine.'"

